# Changes in the Neighborhood Built Environment and Chronic Health Conditions in Washington, DC, in 2014-2019: Longitudinal Analysis

**DOI:** 10.2196/74195

**Published:** 2025-12-10

**Authors:** Quynh C Nguyen, Riki Doumbia, Thu T Nguyen, Xiaohe Yue, Heran Mane, Junaid Merchant, Tolga Tasdizen, Mitra Alirezaei, Pankaj Dipankar, Dapeng Li, Penchala Sai Priya Mullaputi, Amrutha Alibilli, Yulin Hswen, Xin He

**Affiliations:** 1 National Institute of Nursing Research National Institutes of Health Bethesda, MD United States; 2 Urban Studies Program Brown University Providence, RI United States; 3 Department of Epidemiology and Biostatistics University of Maryland School of Public Health College Park, MD United States; 4 Department of Electrical and Computer Engineering Scientific Computing and Imaging Institute University of Utah Salt Lake City, UT United States; 5 Department of Geography and the Environment University of Alabama Tuscaloosa, AL United States; 6 Department of Epidemiology and Biostatistics University of California San Francisco San Francisco, CA United States; 7 College of Computer, Mathematical, and Natural Sciences (CMNS), Artificial Intelligence Interdisciplinary Institute (AIM) University of Maryland College Park, MD United States

**Keywords:** urban development, Google Street View, temporal analysis, health outcomes, computer vision, artificial intelligence

## Abstract

**Background:**

Google Street View (GSV) images offer a unique and scalable alternative to in-person audits for examining neighborhood built environment characteristics. Additionally, most prior neighborhood studies have relied on cross-sectional designs.

**Objective:**

This study aimed to use GSV images and computer vision to examine longitudinal changes in the built environment, demographic shifts, and health outcomes in Washington, DC, from 2014 to 2019.

**Methods:**

In total, 434,115 GSV images were systematically sampled at 100 m intervals along primary and secondary road segments. Convolutional neural networks, a type of deep learning algorithm, were used to extract built environment features from images. Census tract summaries of the neighborhood built environment were created. Multilevel mixed-effects linear models with random intercepts for years and census tracts were used to assess associations between built environment changes and health outcomes, adjusting for covariates, including median age, percentage male, percentage Hispanic, percentage African American, percentage college educated, percentage owner-occupied housing, and median household income.

**Results:**

Washington, DC, experienced a shift toward higher-density housing, with non-single-family homes rising from 66% to 72% of the housing stock. Single-lane roads increased from 37% to 42%, suggesting a shift toward more sustainable and compact urban forms. Gentrification trends were reflected in a rise in college-educated residents (16%-41%), a US $17,490 increase in the median household income, and a US $159,600 increase in property values. Longitudinal analyses revealed that increased construction activity was associated with lower rates of obesity, diabetes, high cholesterol, and cancer, while growth in non-single-family housing was correlated with reductions in the prevalence of obesity and diabetes. However, neighborhoods with higher proportions of African American residents experienced reduced construction activity.

**Conclusions:**

Washington, DC, has experienced significant urban transformation, marked by substantial changes in neighborhood built environments and demographic shifts. Urban development is associated with reduced prevalence of chronic conditions. These findings highlight the complex interplay between urban development, demographic changes, and health, underscoring the need for future research to explore the broader impacts of neighborhood built environment changes on community composition and health outcomes. GSV imagery, along with advances in computer vision, can aid in the acceleration of neighborhood studies.

## Introduction

### Background

Neighborhood characteristics have been identified as critical determinants of various health behaviors and outcomes [1,2]. Previous studies have examined neighborhood walkability, streetscape design, residential density, and land use mix [3-5]. A previous Google Street View (GSV) study analyzing 31 million images found that neighborhoods with more non-single-family homes, an indicator of mixed land use, are associated with lower rates of diabetes and obesity [6]. Another North American study found that a greater land use mix is associated with reduced obesity prevalence [7]. Walkability has been associated with higher physical activity levels, reduced obesity and diabetes risk, and a lower waist-to-hip ratio [8-11]. A study conducted by Carson et al [12] involving 690 participants from 380 census block groups in Baltimore, Seattle, and Washington, DC, metropolitan areas found that increasing neighborhood walkability is associated with increased youth total physical activity [12]. Other studies, both domestic and international, have identified links between built environment characteristics and health outcomes in youth [13-15].

Sidewalks play a critical role in pedestrian safety by providing a designated space separate from vehicular traffic [16]. The risk of pedestrian-vehicle collisions is more than twice as high in areas lacking sidewalks compared to those with them [17]. Sidewalks are particularly important in neighborhoods where children play outside with limited adult supervision [18]. A national investigation [19] by Johns Hopkins University analyzed the effects of lane width on traffic safety across various urban settings and found that lane widths of 10 feet are most appropriate in urban areas, as they balance safety and mobility. Wider lanes (over 11 feet) may lead to unintended speeding and occupy valuable space that could be allocated to other modes of transportation, such as wider sidewalks or bike lanes. These findings suggest that narrower lanes can contribute to traffic calming and improve pedestrian safety [17-19]. The ABQ Streets Project evaluated alternative street designs to retrofit typical residential streets in Albuquerque [20]. The study [20] assessed existing neighborhood design features and proposed modifications, including the addition of sidewalks and the implementation of single-lane roads; the findings suggested that such designs can improve pedestrian safety and accessibility. Collectively, these studies highlight the importance of infrastructural elements to promote safety, accessibility, and livability in urban environments.

Although much of the existing literature has been cross-sectional, offering only a snapshot in time and limiting our ability to assess the dynamic nature of neighborhoods, longitudinal research is essential for identifying how neighborhood changes over time impact health. One longitudinal cohort study (2013-2018) [21] in low-income, predominantly African American Pittsburgh neighborhoods found that enhancements in urban design, such as public art, landscaping, and signage, are linked to reduced wakefulness after sleep onset, highlighting the potential impact of environmental aesthetics on sleep quality. Another longitudinal study [22] found that people living in neighborhoods with high walkability, reduced urban sprawl, and increased recreational access have a lower mean BMI; the meta-analysis of various longitudinal studies further demonstrated that changes in built characteristics, such as enhanced walkability and access to amenities in urban redevelopment, lead to substantial improvements in cardiometabolic health [22].

Although these studies offer valuable insights, many rely on resource-intensive in-person audits or traditional geographic information system (GIS) data, which may lack the spatial granularity or visual detail needed to capture microlevel environmental change. As such, there remains a critical gap in longitudinal neighborhood health research that leverages scalable, low-cost, and reproducible data sources, such as GSV imagery processed with computer vision, to examine built environment changes. Additionally, previous studies have largely relied on cross-sectional surveys, government property records, and satellite-derived geospatial datasets (eg, National Land Cover Database) [23-25]. Although these approaches certainly provide valuable public health surveillance insights, they have critical limitations: self-reported surveys may be subject to bias, official records may lack real-time information, and satellite images may not have street-level granularity. In contrast, GSV offers large-scale, real-time, street-level data with widespread coverage. GSV images reveal reliable physical features that are consistent with topological field assessments [26]. Furthermore, GSV built environment characteristics have been linked with chronic health outcomes by county and zip code levels [27,28].

For researchers interested in neighborhood built environment characteristics and longitudinal changes, GSV images provide a unique data source to examine these characteristics without the need for time-consuming and expensive in-person audits over multiple years. We used GSV images from 2014 to 2019 to quantify longitudinal changes in neighborhood built environments over a 6-year study period in Washington, DC. We selected Washington, DC, due to its rapid urban development and evolving neighborhood characteristics during the study period. Between 2000 and 2013, Washington, DC, was rated as the most intensely gentrifying city in the United States, with approximately 20,000 African Americans displaced from their neighborhoods [29]. Data from 2012 to 2017 ranked the city of Washington, DC, in 13th place, due to gentrification increasing in other US cities [30].

### Study Aims

This study aimed to investigate changes in the built environment in Washington, DC, derived from GSV images. Furthermore, the study examined how changes in built environment characteristics are associated with health outcomes, including the chronic disease burden (eg, obesity, diabetes) and mental health. The study also explored links between urban development and the neighborhood sociodemographic composition, including shifts in racial/ethnic minorities and the median household income.

### Study Hypotheses

We hypothesized the following:

- Urban development increases, as indicated by built environment characteristics derived from GSV images.
- Urban development is associated with a decreased chronic disease burden (obesity, diabetes) and mental distress.
- Changes in the sociodemographic neighborhood composition are associated with changes in the built environment.

## Methods

### Google Street View Data Collection

We collected historical GSV images from Washington, DC, spanning 2014-2019. In total, 434,115 GSV images were collected using the GSV Image application programming interface (API). The GSV API is a tool from Google Maps that allows users to access and download 360° panoramic images of streets worldwide. These images are captured by GSV cars, equipped with advanced cameras, making detailed visual data available. Static images were retrieved by sending API calls with parameters such as year, latitude/longitude coordinates, and camera angle [31]. Images were sampled every 100 m along all primary and secondary roads, with four images captured at each point (facing north, south, east, and west) to create a full view of the built environment.

For longitudinal GSV data collection, we used the Python package *Streetview* [32] to retrieve panorama IDs: unique identifiers for GSV panoramic images, including the year captured. Using these panorama IDs with the GSV Static API, we were able to collect corresponding GSV images for each geocoordinate or its nearest available point. We used the same sampling coordinates for each year of GSV data collection (ie, images were downloaded to capture the same location for each year between 2014 and 2019). However, although we used the same coordinates to sample images each year, image availability could differ depending on whether an updated image was available for any given year of data collection. On average, GSV data were available for 158 census tracts (88% of all census tracts) in Washington, DC, across the years of the study period.

### Built Environment Indicators

We selected neighborhood characteristics that were shown in prior research to influence health outcomes [33] and that could be robustly identified using computer vision models. Additionally, we incorporated less commonly studied indicators, such as stop signs and road construction, to expand the existing neighborhood literature. In total, we examined the following six built environment indicators to represent (1) urban development (non-single-family homes, single-lane roads, construction, two or more cars) and (2) walkability (sidewalks, streetlights, stop signs). In this study, stop signs were used as a measure of intersection density, increased pedestrian safety, and area walkability, all of which are associated with better health outcomes [34]. Road construction was used as a proxy for ongoing urbanization and changes to the built environment. Since residents of sprawling areas tend to live in single-family homes, non-single-family homes were used as an indicator of nearby dense residential and commercial buildings and mixed land use, which have been connected to better health outcomes [6,35]. Single-lane roads were used to indicate residential streets or areas with lower levels of urban development. The presence of two or more cars was used to represent higher traffic density. Sidewalks and streetlights served as walkability indicators, which have been linked to improved health outcomes [36-39].

### Image Data and Quality Control Analyses

Next is a summary of our image analysis; for additional details, please refer to prior publications [40,41]. To create training and test datasets for the computer vision models, 18,000 GSV images from the national data collection were manually annotated. Each image was labeled with a binary “yes/no” value for the presence of six built environment indicators: (1) non-single family homes (single family detached house vs other building type), (2) single-lane roads (one-lane road for motor vehicles vs two or more lanes for traffic demarcated by lines), (3) two or more cars (two or more motor vehicles; some vehicles may be in the background), (4) sidewalks (presence of a sidewalk on at least one side of the road), (5) streetlights (presence of a lamp fixture mounted on a pole spaced along a street or road); and (6) road construction (presence of road construction indicated by orange signs, traffic control devices such as barriers or cones, construction equipment and workers). A value of “yes” indicated the presence of the indicator (eg, sidewalk), while “no” indicated its absence. The principal investigator and three graduate research assistants conducted the data annotation. Interrater agreement was above 85% for all neighborhood indicators. Annotation proceeded in small batches (eg, 100 images per batch). To ensure labeling consistency, the annotators first labeled the same batch, and interrater agreement was calculated for each indicator. Discrepancies were resolved through group discussion and consensus. If 85% interrater reliability was not reached, the labelers were assigned a new batch of images to annotate. Training was iterative until 85% interrater reliability was achieved for each neighborhood indicator, at which point the annotators were asked to label 3000-5000 images independently. These data were then divided into training (80%) and test (20%) datasets.

To generate census tract–level summaries for each GSV-derived built environment characteristic, we used the latitude and longitudinal coordinates associated with each image to assign them to a census tract. Next, for each census tract, we calculated the percentage of the total number of images that contained a given built environment indicator (eg, number of images with a sidewalk/total number of images) × 100 = percentage with sidewalks. We created tertiles and classified each census tract based on its percentage and year of data collection, with the lowest tertile serving as the reference group.

A deep convolutional neural network architecture, Visual Geometry Group (VGG)-19 and Residual Network (ResNet)-18, in TensorFlow was trained with sigmoid cross-entropy with logits as the loss function. These computer vision models were run on 434,115 GSV images from Washington, DC, to produce inferences for the six built environment characteristics. Quality control statistics were as follows: non-single-family homes (accuracy 82%), single-lane roads (accuracy 88%), road construction (accuracy 96%, *F*_1_-score 62%), two or more cars (accuracy 88%, *F*_1_-score 79%), sidewalks (accuracy 84%, *F*_1_-score 81%), streetlights (accuracy 88%, *F*_1_-score 60%), and stop signs (accuracy 90%, *F*_1_-score 89%). Non-single-family homes and single-lane roads were derived from a previous study that did not report *F*_1_-scores [40]. For each year of data, GSV-derived built environment indicators were categorized into tertiles, with the lowest tertile serving as the reference group. This was done to examine potential dose-response associations and to allow for potential changes in the directionality of the associations between tertiles in the built environment characteristics and health outcomes. Using tertiles also allowed comparison with prior GSV-based studies [27,40,42,43]. Sensitivity analyses used linear (continuous) forms of built environment characteristics.

### Census Tract Health Outcomes and Sociodemographic Characteristics

Census tract–level health outcomes data for 2014-2019 were obtained from the Centers for Disease Control and Prevention’s (CDC) PLACES (Population Level Analysis and Community Estimates) program [44]. PLACES is a collaboration between the CDC, the Robert Wood Johnson Foundation, and the CDC Foundation to provide model-based estimates of health measures for census tracts. Additionally, census tract sociodemographic characteristics were obtained from the 1-year estimates of the American Community Survey (ACS) for the period 2014-2019. ACS census tract–level 1-year estimates were also obtained for median property values and median gross rent costs from the database on “Selected Housing Characteristics.” Prior to 2022, the ACS did not adjust home values for inflation, so we did not adjust the values for inflation to preserve the accuracy of the original 1-year estimates.

### Statistical Analysis

We calculated descriptive statistics to examine yearly changes in built environment characteristics, sociodemographics, and health outcomes from 2014 to 2019. Longitudinal analyses were conducted using multilevel mixed-effects linear models for each health outcome, with tertiles for six built environment characteristics as the independent variables of interest. These models included random intercepts for census tracts and random slopes for years (centered on 2016). The six built environment indicators analyzed were single-lane roads, the presence of two or more cars, streetlights, non-single-family homes, sidewalks, and road construction. Models controlled for fixed effects, including census tract, median age, percentage male, percentage Hispanic, percentage African American, percentage college educated, median household income, and percentage owner-occupied housing as covariates. All covariates were standardized to have a mean of 0 (SD 1). We also ran separate multilevel mixed-effects linear models to assess whether changes in neighborhood sociodemographics were associated with changes in built environment characteristics using the same random effect’s structure and covariate adjustments.

### Ethical Considerations

The study was conducted in accordance with the Declaration of Helsinki and was approved by the Institutional Review Board (IRB) of the University of Maryland (protocol number 1074955-8; IRB approval date June 9, 2022).

## Results

### Temporal Trends in the Built Environment in Washington, DC (2014-2019)

**Figure 1:**
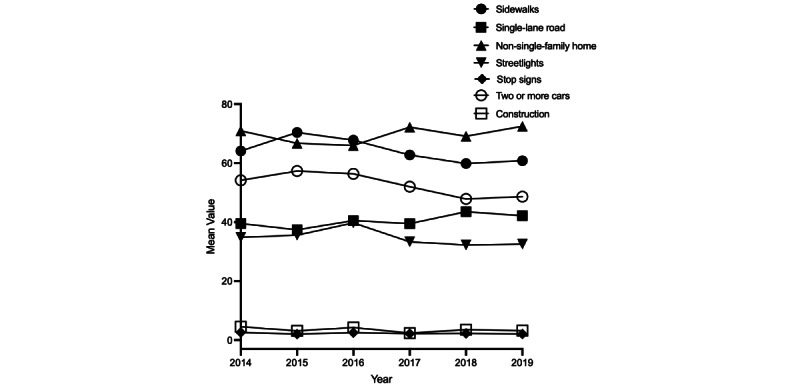
Temporal change in built environment characteristics across census tracts in Washington, DC, 2014-2019.

**Figure 2:**
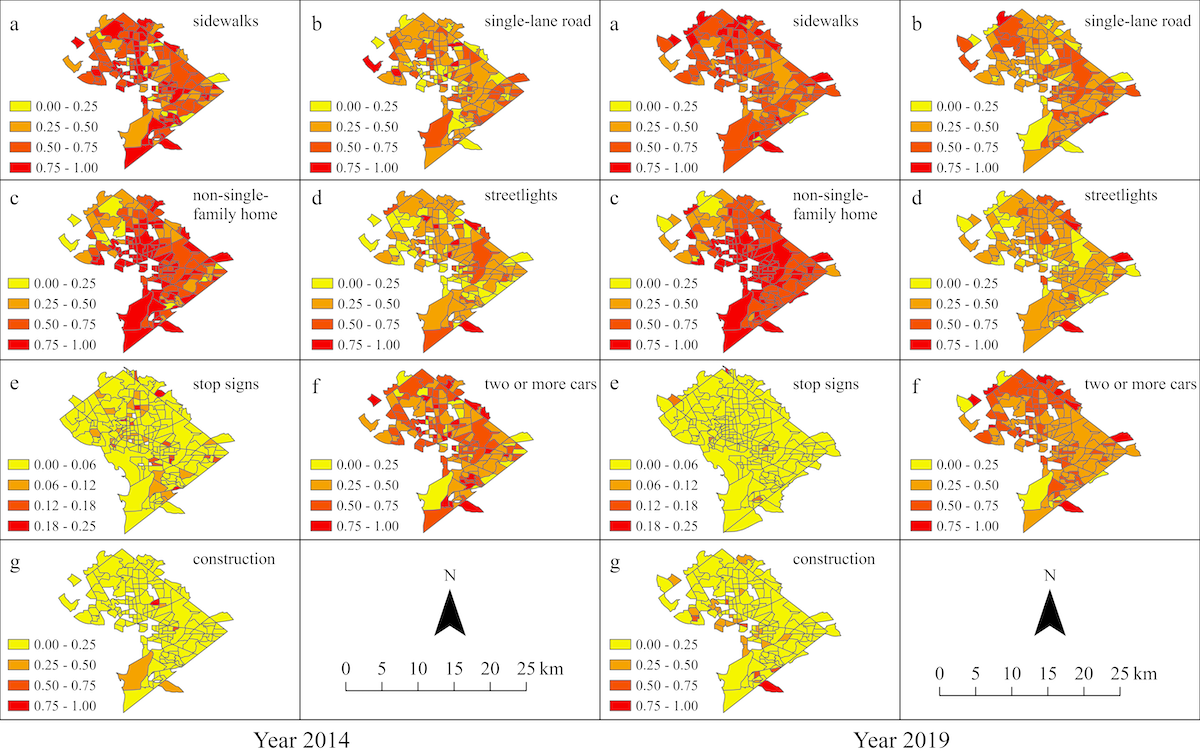
Geographic distribution of built environment characteristics across census tracts in Washington, DC, in 2014 and 2019.

Built environment characteristics changed over the study period, reflecting shifts in the urban landscape of Washington, DC (Figure 1 and Table 1). The proportion of non-single-family homes (a proxy of mixed land use) increased from 70.94% in 2014 to 72.48% in 2019 (Figure 2). The prevalence of single-lane roads rose from 39.53% in 2014 to 42.16% in 2019. Other built environment characteristics (sidewalks, streetlights, two or more cars) increased from 2014 to 2016 and then declined from 2017 to 2019, resulting in lower prevalence rates in 2019 compared to 2014. For instance, the prevalence of sidewalks increased from 2014 (64.12%) to 2016 (67.8%) and then decreased from 2017 to 2019, reaching a prevalence lower than the baseline of 60.83% in 2019. Sensitivity analyses using another GSV-derived walkability indicator (presence of crosswalks) also revealed a similar trend: increasing prevalence between 2014 and 2016, followed by a decrease from 2016 to 2019 (Table S1 in Multimedia Appendix 1). Similarly, streetlight presence increased from 34.85% in 2014 to 39.75% in 2016 but then decreased to 32.55% in 2019. The proportion of two or more cars in GSV images (a proxy of traffic density) increased from 54.19% in 2014 to 56.36% in 2016 but then decreased to 48.65% in 2019 (Figure 2 and Table 1). There was a slight decrease in the prevalence of stop signs, from 2.58% in 2014 to 2.09% in 2019, and a slight decrease in road construction, from 4.54% in 2014 to 3.22% in 2019.

**Table 1:** Descriptive statistics of neighborhood built characteristics, census tract characteristics, and health outcomes in Washington, DC^a^.

Characteristics		Years					
		2014	2015	2016	2017	2018	2019
						
**Built environment characteristics**							
	Single-lane roads (%), mean (SD)	39.53 (21.22)	37.39 (27.74)	40.53 (23.65)	39.50 (22.72)	43.54 (17.21)	42.16 (15.19)
	Stop signs (%), mean (SD)	2.58 (4.82)	2.03 (5.85)	2.51 (5.19)	2.21 (4.07)	2.26 (3.30)	2.09 (1.58)
	Two or more cars (%), mean (SD)	54.19 (23.54)	57.35 (28.31)	56.36 (24.49)	52.00 (27.15)	47.85 (18.23)	48.65 (15.81)
	Streetlights (%), mean (SD)	34.85 (21.10)	35.57 (27.04)	39.75 (24.76)	33.33 (22.29)	32.26 (15.56)	32.55 (13.90)
	Non-single-family homes (%), mean (SD)	70.94 (24.35)	66.74 (31.14)	66.01 (27.19)	72.19 (26.78)	69.12 (22.93)	72.48 (19.86)
	Sidewalks (%), mean (SD)	64.12 (24.16)	70.39 (28.79)	67.80 (23.66)	62.77 (27.89)	59.87 (18.76)	60.83 (15.50)
	Road construction (%), mean (SD)	4.54 (10.90)	3.16 (10.21)	4.28 (11.09)	2.39 (5.35)	3.55 (5.53)	3.22 (3.94)
	Census tracts, n	162	143	152	155	166	169
**Census tract characteristics**							
	Population size (n), mean (SD)	3405 (1370)	3418 (1382)	3428 (1399)	3457 (1382)	3463 (1386)	3468 (1390)
	Median age (years), mean (SD)	35.24 (6.79)	35.02 (6.34)	34.93 (6.08)	34.84 (6.00)	34.82 (5.76)	34.79 (5.67)
	Percentage male (%), mean (SD)	47.25 (6.10)	47.23 (6.22)	47.47 (5.89)	47.59 (5.88)	47.51 (5.77)	47.46 (5.66)
	Percentage African American (%), mean (SD)	55.06 (35.14)	53.87 (34.97)	52.77 (34.68)	51.85 (34.59)	51.11 (34.40)	50.15 (34.19)
	Percentage Hispanic (%), mean (SD)	8.76 (8.64)	9.17 (8.61)	9.59 (8.42)	9.89 (8.33)	10.08 (8.44)	10.28 (8.22)
	Median household income (US $), mean (SD)	74,026 (41,041)	75,466 (40,972)	78,360 (43,295)	82,936 (45,084)	86,504 (44,706)	91,516 (46,229)
	Percentage college educated (%), mean (SD)	15.82 (9.34)	15.92 (9.14)	16.17 (9.27)	16.78 (9.15)	40.05 (24.43)	40.96 (24.29)
	Percentage owner-occupied housing (%), mean (SD)	38.35 (20.54)	38.74 (21.12)	38.25 (21.39)	38.65 (20.96)	38.99 (20.85)	39.04 (21.16)
	Census tracts, n	178	178	178	178	178	178
**Adult health outcomes**							
	Obesity (%), mean (SD)	27.40 (7.74)	27.78 (8.01)	28.01 (7.86)	28.34 (8.14)	30.00 (7.95)	26.95 (7.85)
	Diabetes (%), mean (SD)	10.18 (4.49)	10.37 (4.54)	9.70 (4.21)	10.15 (4.44)	10.70 (4.63)	9.20 (4.04)
	High blood pressure (%), mean (SD)	31.53 (10.18)	31.53 (10.18)	31.28 (9.98)	31.65 (9.66)	32.14 (9.79)	32.14 (9.79)
	High cholesterol (%), mean (SD)	32.02 (5.39)	32.02 (5.39)	28.93 (4.91)	29.34 (4.93)	27.73 (4.77)	27.73 (4.77)
	Cancer (%), mean (SD)	5.18 (1.48)	4.90 (1.41)	4.99 (1.43)	5.71 (1.62)	5.62 (1.62)	5.10 (1.56)
	Poor mental health days (%), mean (SD)	12.17 (3.82)	11.73 (3.55)	12.11 (3.79)	14.04 (4.16)	13.78 (3.91)	15.38 (3.25)
	Census tracts, n	154	154	154	174	174	174

^a^Data sources: (1) Google Street View (GSV) built environment characteristics, (2) American Community Survey (ACS) census tract sociodemographic characteristics, and (3) Centers for Disease Control (CDC) Prevention Population Level Analysis and Community Estimates (PLACES) health outcomes.

Figure 2 displays the geographic distribution of GSV-derived built environment characteristics across census tracts in Washington, DC, and across time. Stop signs appeared more evenly distributed across Washington, DC, in 2014 than in 2019, while the converse was true for road construction, which seemed more dispersed in 2019 than in 2014. Non-single-family homes gained even more prominence over time, except in the northwestern parts of Washington, DC. The presence of two or more cars on city streets decreased over time, especially in the southern areas of Washington, DC.

### Changes in Property Values, Housing Costs, and Sociodemographics in Washington, DC

**Figure 3:**
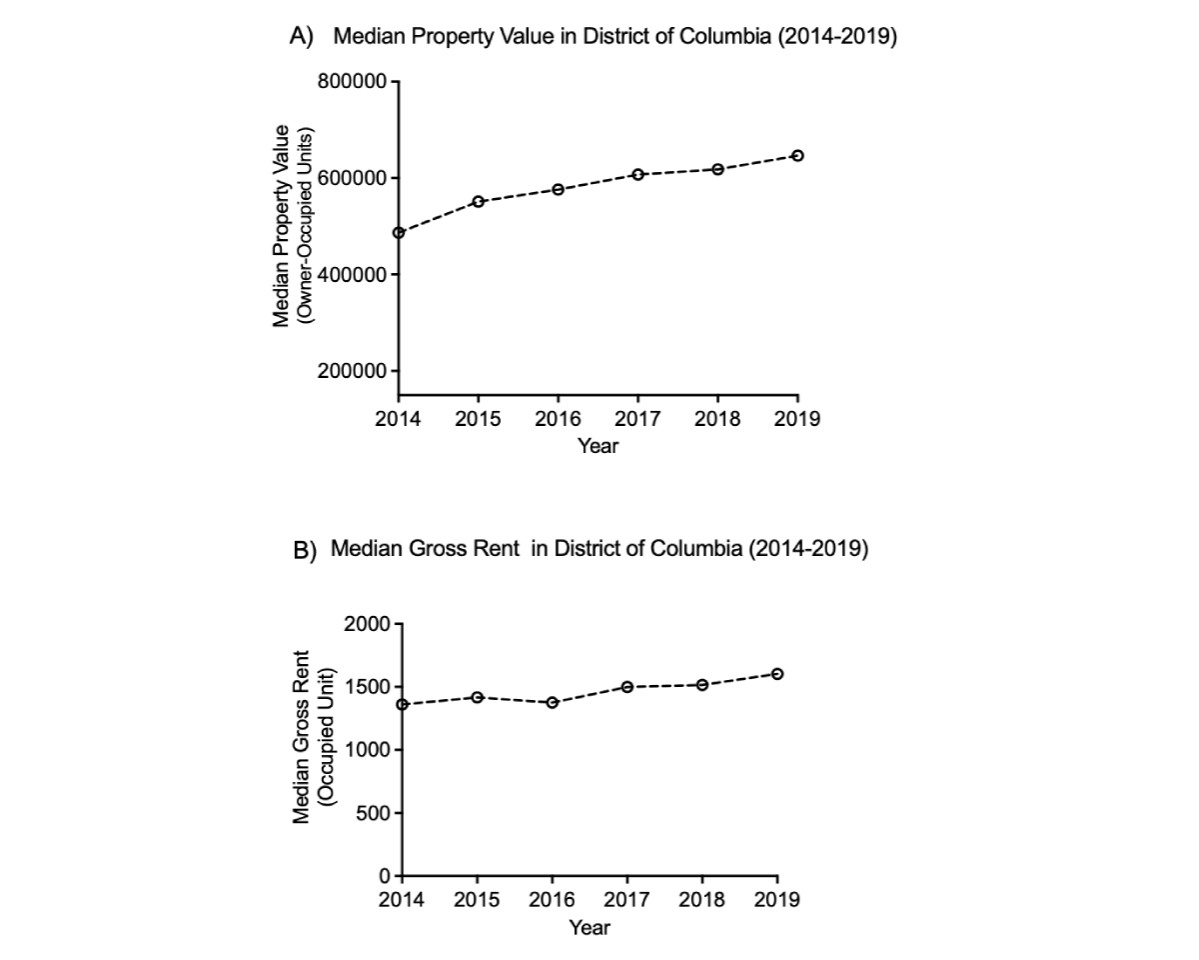
Housing prices in Washington, DC, 2014-2019: (A) median property values and (B) median gross rent costs. Data source: American Community Survey.

Over the study period, median property values in Washington, DC, rose significantly, increasing from US $486,900 in 2014 to US $646,500 in 2019 (a difference of US $159,600; Figure 3A). Similarly, median monthly rent costs increased by almost 20%, rising from US $1360 in 2014 to US $1603 in 2019 (Figure 3B).

Dramatic shifts occurred in socioeconomic characteristics, with the percentage of college-educated residents increasing from 15.83% to 40.96% and the median household income increasing by US $17,490 over these 6 years (Table 1). The proportion of Washington, DC, residents who are African American declined from 55.06% in 2014 to 50.15% in 2019, while the Hispanic population increased from 8.76% to 10.28% during the same period (Table 1). The median age, owner-occupied housing rates, and the percentage of male residents remained relatively stable (Table 1).

### Changes in Health Outcomes in Washington, DC

High cholesterol rates steadily declined from 32.02% in 2014 to 27.73% in 2019 (Table 1). Cancer prevalence was highest in 2017 at 5.71% and then declined to 5.1% in 2019. High blood pressure was relatively stable, with a prevalence of 31.53% in 2014 and 32.14% in 2019. Obesity rates showed a consistent upward trend until 2018, before dropping by ~3% in 2019. A similar trend was seen for diabetes, which saw a high of 10.7% in 2018, followed by a decline in 2019 (9.2%). Poor mental health days increased steadily throughout the study period from 12.17% in 2014 to 15.38% in 2019.

### Longitudinal Analyses

Census tracts with the highest proportion of non-single-family homes (ie, mixed land use) experienced a 0.39% decrease in obesity and a 0.2% decrease in diabetes rates compared to those with the lowest proportion of non-single-family homes (Table 2). Increased road construction activity was associated with a 0.91% decrease in obesity rates and a 0.41% decrease in diabetes rates, comparing the second tertile to the first. Road construction activity in the highest tertile was associated with a 0.17% decrease in high cholesterol prevalence compared to the lowest tertile. Contrary to initial hypotheses, sidewalks had mixed associations (Tables 2 and 3). An increase in stop signs was linked with a reduction in high cholesterol (second tertile only) and marginally with a decrease in obesity but was associated with an increase in poor mental health days (Tables 2 and 3). Higher proportions of African American residents were associated with an increased prevalence of chronic conditions and poor mental health days (Table 3), with an SD increase in the African American population associated with a 6.81% rise in obesity (Table 2). A higher proportion of owner-occupied housing was associated with reduced rates of chronic diseases and poor mental health days (Table 3). The median household income was associated with reduced prevalence of obesity, diabetes, and high cholesterol (Tables 2 and 3).

**Table 2:** Built environment predictors of adult health outcomes (obesity, diabetes, high blood pressure)^a^.

Predictors		Obesity	Diabetes	High blood pressure
		Change % (95% CI)^b^	Change % (95% CI)^b^	Change % (95% CI)^b^
Year (centered 2016)		0.16 (0.08 to 0.24)^c^	–0.05 (–0.10 to 0.00)^c^	0.11 (0.05 to 0.17)^c^
**Single-lane roads**				
	Third tertile (highest)	0.26 (–0.05 to 0.57)	0.09 (–0.08 to 0.27)	0.05 (–0.08 to 0.18)
	Second tertile	0.11 (–0.15 to 0.37)	0.07 (–0.08 to 0.21)	0.03 (–0.07 to 0.14)
**Stop signs**				
	Third tertile (highest)	–0.20 (–0.44 to 0.04)^d^	–0.03 (–0.17 to 0.10)	0.07 (–0.03 to 0.17)
	Second tertile	–0.31 (–0.66 to 0.04)^d^	–0.02 (–0.22 to 0.18)	0.07 (–0.07 to 0.22)
**Two or more cars**				
	Third tertile (highest)	–0.22 (–0.57 to 0.14)	–0.15 (–0.35 to 0.05)^d^	–0.18 (–0.33 to –0.04)^c^
	Second tertile	–0.19 (–0.48 to 0.10)	–0.15 (–0.32 to 0.01)	–0.07 (–0.19 to 0.05)
**Streetlights**				
	Third tertile (highest)	0.09 (–0.21 to 0.38)	0.06 (–0.10 to 0.23)	0.00 (–0.12 to 0.13)
	Second tertile	–0.04 (–0.31 to 0.22)	0.01 (–0.13 to 0.16)	0.09 (–0.02 to 0.19)
**Non-single-family homes**				
	Third tertile (highest)	–0.39 (–0.74 to –0.03)^c^	–0.20 (–0.39 to 0.00)	–0.11 (–0.26 to 0.03)
	Second tertile	–0.15 (–0.44 to 0.14)	–0.13 (–0.30 to 0.03)	–0.04 (–0.16 to 0.08)
**Sidewalks**				
	Third tertile (highest)	0.20 (–0.16 to 0.56)	0.12 (–0.08 to 0.32)	0.06 (–0.08 to 0.21)
	Second tertile	0.34 (0.06 to 0.61)^c^	0.12 (–0.03 to 0.28)	0.02 (–0.09 to 0.14)
**Road construction**				
	Third tertile (highest)	–0.01 (–0.24 to 0.23)	0.00 (–0.13 to 0.13)	0.06 (–0.03 to 0.16)
	Second tertile	–0.91 (–1.34 to –0.48)^c^	–0.41 (–0.66 to –0.16)^c^	0.19 (–0.01 to 0.38)^d^
**Census tract sociodemographics**				
	Percentage male	0.00 (–0.20 to 0.21)	–0.12 (–0.24 to 0.01)	–0.10 (–0.23 to 0.02)
	Median age	–0.19 (–0.46 to 0.08)	0.35 (0.18 to 0.52)^c^	0.09 (–0.08 to 0.26)
	Percentage African American	6.81 (6.34 to 7.27)^c^	3.74 (3.44 to 4.04)^c^	3.85 (3.27 to 4.43)^c^
	Percentage Hispanic	0.68 (0.42 to 0.95)^c^	0.41 (0.24 to 0.57)^c^	0.61 (0.42 to 0.79)^c^
	Percentage college educated	0.51 (0.31 to 0.71)^c^	0.23 (0.12 to 0.34)^c^	0.31 (0.21 to 0.40)^c^
	Median household income	–0.75 (–1.17 to –0.32)^c^	–0.49 (–0.75 to –0.24)^c^	–0.72 (–0.97 to –0.47)^c^
	Percentage owner-occupied housing	–0.80 (–1.17 to –0.42)^c^	–0.11 (–0.35 to 0.13)	–0.31 (–0.64 to 0.02)^d^

^a^Data source for health outcomes: Centers for Disease Control and Prevention (CDC) Population Level Analysis and Community Estimates (PLACES).

^b^Multilevel mixed-effects linear models were run for each outcome separately with random intercepts for census tract levels (153 tracts) and random slopes for years (centered 2016). Models controlled for census tract median age, percentage male, percentage Hispanic, percentage African American, percentage with a college degree, median household income, and percentage owner-occupied housing. Built environment characteristics were categorized into tertiles, with the lowest tertile serving as the reference group. Census tract sociodemographic characteristics were standardized with a mean of 0 (SD 1).

^c^*P*<.05.

^d^*P*<.10.

**Table 3:** Built environment predictors of adult health outcomes (high cholesterol, cancer, mental health days)^a^.

Predictors		High cholesterol	Cancer	Mental health days
		Change % (95% CI)^b^	Change % (95% CI)^b^	Change % (95% CI)^b^
Year (centered 2016)		–1.16 (–1.23 to –1.09)^c^	0.07 (0.05 to 0.09)^c^	0.72 (0.65 to 0.79)^c^
**Single-lane roads**				
	Third tertile (highest)	0.11 (–0.11 to 0.33)	–0.03 (–0.10 to 0.05)	0.08 (–0.15 to 0.32)
	Second tertile	0.03 (–0.15 to 0.21)	–0.02 (–0.08 to 0.05)	0.13 (–0.06 to 0.33)
**Stop signs**				
	Third tertile (highest)	–0.11 (–0.28 to 0.05)	0.04 (–0.02 to 0.10)	0.30 (0.12 to 0.48)^c^
	Second tertile	–0.25 (–0.51 to 0.00)^c^	–0.03 (–0.12 to 0.05)	0.25 (–0.02 to 0.51)^d^
**Two or more cars**				
	Third tertile (highest)	–0.04 (–0.29 to 0.21)	0.07 (–0.02 to 0.15)	0.13 (–0.14 to 0.40)
	Second tertile	–0.06 (–0.27 to 0.14)	0.03 (–0.04 to 0.10)	0.05 (–0.17 to 0.27)
**Streetlights**				
	Third tertile (highest)	0.04 (–0.17 to 0.24)	–0.03 (–0.10 to 0.04)	0.05 (–0.18 to 0.27)
	Second tertile	0.14 (0.04 to 0.33)	–0.02 (–0.08 to 0.04)	0.18 (–0.02 to 0.38)^d^
**Non-single-family homes**				
	Third tertile (highest)	0.13 (–0.12 to 0.38)	–0.01 (–0.09 to 0.08)	0.18 (–0.09 to 0.45)
	Second tertile	0.03 (–0.18 to 0.23)	–0.01 (–0.08 to 0.06)	0.14 (–0.08 to 0.35)
**Sidewalks**				
	Third tertile (highest)	0.26 (0.01 to 0.52)^c^	–0.08 (–0.16 to 0.01)^d^	–0.21 (–0.48 to 0.06)
	Second tertile	–0.07 (–0.26 to 0.12)	–0.04 (–0.11 to 0.03)	–0.21 (–0.41 to 0.00)^c^
**Road construction**				
	Third tertile (highest)	–0.17 (–0.33 to –0.01)^c^	–0.04 (–0.09 to 0.02)	0.01 (–0.17 to 0.18)
	Second tertile	0.21 (–0.10 to 0.53)	–0.10 (–0.20 to 0.00)^c^	0.17 (–0.17 to 0.51)
**Census tract sociodemographics**				
	Percentage male	–0.21 (–0.39 to –0.03)^c^	–0.01 (–0.06 to 0.04)	0.10 (–0.06 to 0.27)
	Median age	0.42 (0.18 to 0.67)^c^	0.08 (0.01 to 0.15)^c^	–0.57 (–0.79 to –0.36)^c^
	Percentage African American	3.01 (2.46 to 3.57)^c^	0.07 (–0.11 to 0.25)	2.45 (2.08 to 2.82)^c^
	Percentage Hispanic	0.67 (0.42 to 0.93)^c^	–0.02 (–0.10 to 0.05)	0.16 (–0.05 to 0.36)
	Percentage college educated	0.61 (0.46 to 0.76)^c^	–0.05 (–0.10 to 0.00)^c^	0.05 (–0.11 to 0.20)
	Median household income	0.23 (–0.13 to 0.59)	0.01 (–0.10 to 0.11)	–0.14 (–0.49 to 0.20)
	Percentage owner-occupied housing	0.07 (–0.34 to 0.48)	0.29 (0.16 to 0.42)^c^	–1.13 (–1.42 to –0.83)^c^

^a^Data source for health outcomes: Centers for Disease Control and Prevention Population Level Analysis and Community Estimates (CDC PLACES).

^b^Multilevel mixed-effects linear models were run for each outcome separately with random intercepts for census tract levels (153 tracts) and random slopes for years (centered 2016). Models controlled for census tract median age, percentage male, percentage Hispanic, percentage African American, percentage with a college degree, median household income, and percentage owner-occupied housing. Built environment characteristics were categorized into tertiles, with the lowest tertile serving as the reference group. Census tract sociodemographic characteristics were standardized with a mean of 0 (SD 1).

^c^*P*<.05.

^d^*P*<.10.

In sensitivity analyses, we implemented regression analyses using continuous (linear forms) variables of the built environment characteristics rather than tertiles (Table S2 in Multimedia Appendix 1). Results from the linear models were generally consistent with the tertile-based analyses. However, some associations did not reach statistical significance in regression models with linear forms of the built environment characteristics. For instance, although the second tertile of sidewalks was positively associated with obesity and negatively linked with poor mental health days compared to the first tertile, these associations were not statistically significant when sidewalks were modeled as a continuous variable. Additionally, some associations that were statistically significant in the third tertile (compared to the first tertile) were not statistically significant in the linear form of the variable (ie, non-single-family homes and diabetes; construction and high cholesterol). This divergence in patterns detected could stem from tertiles allowing for nonlinear associations and the examination of dose-response associations.

Table 4 shows the results of analyses examining the relationship between changes in neighborhood sociodemographics and the built environment. A 1 SD increase in the proportion of African American residents was linked with a 1.41% decrease in road construction activity and a marginally significant reduction in non-single-family homes, a proxy for mixed land use. A 1 SD increase in the median age was linked to a 4.94% decrease in non-single-family homes and a 0.86% decrease in road construction activity, suggesting lower urban development in census tracts with older populations. In contrast, a 1 SD increase in owner-occupied housing was associated with a 4.1% reduction in non-single-family homes, suggesting that neighborhoods that had higher owner occupancy were less likely to be mixed land use (Table 4). A heatmap of correlations with 2019 data are presented in Figure S1 in Multimedia Appendix 1. Directionality of associations was similar. For instance, the median age was negatively correlated with non-single-family homes (r=–0.51; Figure S1 in Multimedia Appendix 1).

**Table 4:** Sociodemographic predictors of built environment changes^a^.

Predictors			Percentage non-single-family homes (%), change (95% CI)		Percentage road construction (%), change (95% CI)	Percentage sidewalks (%), change (95% CI)
Year (centered 2016)			0.33 (–0.56 to 1.22)		–0.56 (–0.95 to –0.17)^b^	–1.36 (–2.39 to –0.32)^b^
**Census tract sociodemographics**						
	Percentage male (%)	3.39 (1.35 to 5.43)^b^		0.40 (–0.31 to 1.11)		–0.72 (–2.54 to 1.11)
	Median age (%)	–4.94 (–7.44 to –2.43)^b^		–0.86 (–1.66 to –0.06)^b^		–1.14 (–3.17 to 0.89)
	Percentage African American (%)	–3.95 (–8.05 to 0.15)^c^		–1.41 (–2.81 to –0.02)^b^		–2.39 (–5.96 to 1.19)
	Percentage Hispanic (%)	–0.70 (–3.03 to 1.63)		–0.05 (–0.78 to 0.68)		2.06 (0.20 to 3.92)^b^
	Percentage college educated (%)	2.18 (–0.16 to 4.52)^c^		0.83 (–0.17 to 1.83)		–0.44 (–3.05 to 2.17)
	Median household income (%)	–3.19 (–7.51 to 1.13)		–1.37 (–2.87 to 0.12)^c^		–2.98 (–6.79 to 0.84)
	Percentage owner-occupied housing (%)	–4.10 (–7.38 to –0.83)^b^		0.34 (–0.70 to 1.38)		2.59 (–0.07 to 5.24)^c^

^a^Multilevel mixed-effects linear models were run for each built environment characteristic separately with random intercepts for census tract levels (153 tracts) and random slopes for years (centered 2016). Models controlled for the census tract median age, percentage male, percentage Hispanic, percentage African American, percentage college educated, and percentage owner-occupied housing.

^b^*P*<.05.

^c^*P*<.10.

## Discussion

### Changes in the Neighborhood Environment

During the period from 2014 to 2019, Washington, DC, experienced significant urban transformation, marked by substantial changes in neighborhood built environments and demographic shifts. The urban landscape shifted toward higher-density housing, with non-single-family homes rising from 66.01% to 72.48% of the housing stock. The observed growth in non-single-family housing aligns with large-scale mixed-use developments, such as CityCenterDC, the Wharf, and the Anacostia Riverwalk Trail. In addition, the prevalence of single-lane roads increased from 37.39% to 42.16%, indicating a shift toward more sustainable and compact urban forms, which promote slower speeds, reduce car dependency, and increase walkable neighborhoods. Furthermore, other indicators of walkability, such as sidewalks and streetlights, increased from 2014 to 2016. This particularly aligns with early efforts to help improve walkability through multiple initiatives, such as Vision Zero DC (2014) and the Pedestrian Master Plan. However, later declines in walkability indicators contrast with the goals that were laid out according to these plans (Vision Zero DC, Pedestrian Master Plan).

Moreover, during this time period, Washington, DC, underwent rapid gentrification. The median household income increased by US $17,000, reflecting the influx of wealthier residents, and the percentage of the population with a college education increased from 15.82% to 40.96%. Meanwhile, the percentage of African American residents decreased from 55.06% to 50.15%. The decline in the proportion of the African American population, alongside rising income and education levels, is an indicator of gentrification, but it also raises some concerns about racial displacement and equity in the context of urban development [45]. Hwang et al [46] found that gentrification is not strongly associated with increased displacement of lower-income residents; however, the authors found different location outcomes by race, suggesting that racial stratification in the housing market has a dominant effect regardless of gentrification [46].

### Built Environment and Health

The longitudinal analysis performed in this study showed associations with changes in the built environment and various health outcomes in Washington, DC, that provide insights into the possible mechanisms through which urban development can influence population health. One of the important findings of this study is that an increase in non-single-family homes is linked with lower prevalence of obesity and diabetes. This is consistent with prior research analyzing 1.4 million GSV images integrated with electronic health records in Utah, which showed that residents in neighborhoods with more non-single-family homes had a 10%-27% lower prevalence of diabetes, uncontrolled diabetes, hypertension, and obesity [27]. In addition to physical health benefits, prior research has also demonstrated associations between greater land use mix and mental health; a 2021 systematic review [47] found that greater land use mix is associated with decreased psychological distress. Non-single-family homes, typically located in high-population-density areas and mixed-use urban areas, are close to shops and workplaces and promote the use of public transportation; this encourages walking and cycling as part of daily routines. As a result, active transportation in these neighborhoods leads to greater walkability and physical activity than in car-dependent suburbs dominated by single-family homes. In addition, the residents of high-density areas have access to parks, community centers, and recreational facilities that promote outdoor physical activity [48].

Our results align with prior research suggesting that built environment improvements can yield measurable health benefits. For instance, Nguyen et al [42] used GSV imagery to assess neighborhood characteristics and reported that areas classified as more rural or underdeveloped exhibit higher rates of obesity, diabetes, poor self-rated health, and premature mortality, underscoring the link between urban development and population health. Building on this, the health benefits observed in our study may be indicative of well-implemented urbanization programs that enhance access to amenities and services through new construction projects. We found that an increase in road construction is associated with decreased prevalence of obesity, diabetes, high cholesterol, and cancer. These developments can facilitate healthy behavioral changes by preserving or increasing opportunities for physical activity, particularly in areas affected by road closures [49]. For instance, a prior study [50] has shown that when roads are closed to vehicular traffic, residents often utilize the space for recreation.

Furthermore, we identified that the presence of stop signs is associated with a decrease in chronic conditions. Although these associations do not imply causality, they may reflect well-developed urban environments that support healthier lifestyles. This interpretation is partially supported by previous cross-sectional studies that have linked dense areas and traffic-calming measures, such as traffic lights and stop signs, with reduced vehicle speeds and traffic volumes, enhanced safety, and improved quality of life [51,52]. Previous research also suggests that neighborhoods with safer traffic environments and a variety of destinations are more likely to promote physical activity, which can contribute to better health outcomes [53].

In this study, sidewalks were positively associated with obesity but negatively linked with poor mental health days, but these findings were limited to the second tertile versus the first tertile and were not significant when modeled as a continuous variable. Previous studies also indicate a highly complex relationship between walkability indicators and various chronic conditions. For example, Keralis et al [6] analyzed 31 million GSV images and highlighted an increased prevalence of obesity, diabetes, and inactivity associated with the second tertile of crosswalk scores in contrast with a decreased prevalence associated with the third tertile. However, when modeling crosswalks as a continuous linear variable, there was a significant negative correlation between crosswalks and chronic conditions. These mixed findings illustrate the complexity of correlating walkability indicators with health outcomes. On the one hand, sidewalks are associated with urbanicity, where there is a higher prevalence of chronic conditions [54]; on the other hand, they are associated with increased physical activity, which can reduce the risk of negative health outcomes [55]. These mixed findings underscore that sidewalks are proxies for multiple contextual factors, including urban density and opportunities for physical activity. Therefore, the varying associations of sidewalks and health outcomes warrant further investigation and increased efforts to adjust for potential confounding.

Finally, our analyses revealed that neighborhoods with higher proportions of African American residents are associated with reduced road construction and marginally fewer non-single-family homes. This is consistent with gentrification patterns reported for Washington, DC. This is only partially addressed by equity frameworks in transportation plans, such as MoveDC (Washington Post Gentrification Report, MoveDC Equity Framework). Nicoletti et al [56] also reported that urban communities with larger shares of racial minorities, lower income, and less educational attainment face persistent disparities in spatial access to infrastructure. These findings underscore the need to address structural inequities in infrastructure investment and urban planning.

Structural factors have profoundly shaped the housing market in Washington, DC, producing persistent racial disparities in homeownership, property values, and housing access. Beginning in the 1930s, the Homeowners’ Loan Corporation (HOLC) established “Residential Security Maps.” Neighborhoods were assigned a letter grade from A (safest investment) to D (hazardous), with different colors indicating their level of risk for lenders. African American neighborhoods were consistently outlined in red (“hazardous”). This practice of redlining denied residents access to home loans, business capital, and construction funds, thereby entrenching long-term disinvestment [57-60]. In the mid-20th century, “urban renewal” programs further displaced thousands of African American residents [61]. Entire neighborhoods were demolished to make way for commercial development and highways to the suburbs. The city offered little or no compensation or relocation assistance. Following these demolitions, the city concentrated public housing projects in the eastern part of the district, particularly east of the Anacostia River, deepening patterns of racial and economic segregation [62]. These historical policies and practices shaped modern-day neighborhoods in Washington, DC. Today, Washington, DC, remains one of the most racially segregated cities in the United States, with distinct east-west divisions [63]. These housing and planning practices not only entrenched segregation but also restricted access to health-promoting resources, such as quality housing, neighborhood amenities, education, and health care [64,65].

### Strengths of the Study

This study is one of the few to use a longitudinal study design to examine the potential impact of changing built environments, neighborhood sociodemographics, and health outcomes. In addition, we uniquely leveraged innovative data sources and computer vision methods to enable the analysis of millions of street view scenes in Washington, DC. The use of publicly available GSV images offers a useful case study for conducting cost-effective, large neighborhood studies with data not typically included in public health studies. We collected and analyzed four million GSV images to assess changes in neighborhood built environments over 6 years. Neighborhood studies have traditionally relied on costly and time-intensive neighborhood audits or neighborhood surveys [66,67]. Alternatively, other research teams have also set up specially designed camera equipment on vehicles to enable photo data collection during drives through neighborhoods [68]. However, the cost of renting or purchasing that equipment can be prohibitively expensive, in addition to the expertise and staff time needed for operating the equipment. Moreover, primary data collection is often feasible for studies involving certain neighborhoods within a city and is less feasible for comprehensively capturing images across multiple cities or states or conducting national neighborhood studies.

### Limitations of the Study

Nonetheless, our study is subject to some notable limitations. Focusing on a single city may limit statistical power and reduce external validity, as findings may not fully extend to other urban contexts with different social, economic, or planning structures. Future multicity analyses will be important for testing the robustness of these detected patterns and enhancing their policy relevance. Washington, DC, was chosen as a case example, given its well-known gentrification and urban development happening in recent years in the city and to investigate links between built environment changes, sociodemographic changes, and health outcomes using a longitudinal design.

In this study, health outcomes were analyzed separately. In future studies, if the relationships between multiple outcome variables are of interest, multivariate multilevel mixed-effects models can be applied by including additional random effects for multiple correlated outcomes. Additionally, although this study used built environment indicators theoretically and empirically supported by the literature as being important to health (eg, indicators of walkability, such as sidewalks), future studies may incorporate additional features of the neighborhood environment, such as environmental hazards, recreational resources, and indicators of dilapidation.

Moreover, our image data collection was limited by the coverage of GSV for Washington, DC, for our study period. Although the same sampling coordinates were used to collect GSV images for each year, the availability of yearly image updates differed across areas in Washington, DC. GSV coverage data ranged from 143 to 169 of the 178 census tracts in Washington, DC, from 2014 to 2019. Health outcome estimates from CDC PLACES were available for approximately 154 tracts in 2014-2016 and up to 174 tracts in subsequent years.

Partial GSV coverage likely reflects uneven update patterns across neighborhoods, wherein areas with greater commercial activity or higher traffic volumes are prioritized for imagery updates, whereas predominantly residential or lower-income areas may experience reduced update frequency or lower-resolution coverage. This limitation has been noted in prior studies using GSV imagery for neighborhood health research [69]. Similarly, the incomplete availability of health estimates in earlier years likely reflects methodological constraints, including the evolving nature of small-area estimation models and the exclusion of tracts with insufficient population sizes or high uncertainty. As the modeling approach matured and incorporated more robust data sources, coverage improved in later years [70]. These gaps in coverage could have contributed to bias in the analyses. Variation in image update availability could lead to noncorrespondence between changes in the prevalence of a feature in imagery and the true change of that feature in the real world. Nonetheless, the overall data coverage was still high, ranging from 88%-92% of census tracts in Washington, DC.

In addition, CDC PLACES uses a multilevel regression and poststratification (MRP) method to generate estimates for places with smaller populations, such as census tracts and ZIP Code Tabulation Areas. These model-based datasets provide a more granular and comprehensive view of community health than traditional surveillance data, enabling detailed analysis of chronic diseases, preventive services use, health risk behaviors, disabilities, and health-related social needs at the local level [44,71]. However, reliance on modeled health estimates (rather than direct health data) could have introduced error or bias in the analysis.

To derive sociodemographic information, we used the 1-year estimates from the ACS data instead of the 5-year estimates. Although 1-year estimates offer more precise year-by-year demographic snapshots, they come with trade-offs in accuracy due to smaller sample sizes. The 1-year estimate provides estimates for geographic areas with populations of 65,000 or more, and the smaller sample size could result in less precision. Future studies could consider using 5-year estimates as a supplement for greater precision. Despite these limitations, the analytic sample ultimately encompassed approximately 88% of census tracts in Washington, DC, representing substantial spatial coverage and supporting the generalizability of study findings across DC.

### Conclusion

This study used GSV images and computer vision to examine changes in neighborhood built environments, demographics, and health outcomes in Washington, DC, from 2014 to 2019. By analyzing over 430,000 images, we assessed urban development and walkability indicators at the census tract level. We observed notable shifts in the demographic composition of residents and concurrent changes in neighborhood built environments and health outcomes. These patterns underscore the critical connections between urban policy and public health. Key findings showed that increased construction activity and non-single-family housing are associated with reductions in the prevalence of obesity and diabetes, highlighting the potential health benefits of urban development. In contrast, walkability indicators revealed mixed associations: sidewalks were unexpectedly linked to higher obesity and cholesterol rates but to lower rates of poor mental health. Neighborhoods with larger African American populations saw less infrastructure investment, highlighting ongoing racial disparities in urban development. By integrating longitudinal imagery with public health data, this study demonstrates the feasibility of using scalable, low-cost digital tools to monitor neighborhood change and its health impacts. These findings contribute to urban health research and underscore the need for more equitable infrastructure planning. As issues such as gentrification, zoning, residential location in proximity to environmental hazards, and walkability remain at the forefront of urban discourse, it is imperative that future studies investigate the connections between these indicators and health outcomes.

## Data Availability

Population Level Analysis and Community Estimates 2021 and the American Community Survey are publicly available. Google Street View neighborhood-level data can be accessed online [72].

## Appendix

Multimedia Appendix 1 Time trends in walkability indicators in census tracts across Washington DC, 2014-2019; correlations between built environment characteristics and census tract characteristics, 2019; built environment predictors of adult health outcomes.

## Abbreviations

ACS: American Community Survey
API: application programming interface
CDC: Centers for Disease Control and Prevention
GSV: Google Street View
PLACES: Population Level Analysis and Community Estimates
