# Supplementary material for: Changes in the Neighborhood Built Environment and Chronic Health Conditions in Washington, DC, in 2014-2019: Longitudinal Analysis
Source: JMIR Form Res. 2025 Dec 10;9:e74195. doi: 10.2196/74195 (PMC12739454; doi:10.2196/74195)
Supplement: Multimedia Appendix 1 [file formative_v9i1e74195_app1.docx]

**Multimedia Appendix 1**

| **Table S1.** Time trends in walkability indicators in census tracts across Washington DC, 2014-2019. | | | | | | |
| --- | --- | --- | --- | --- | --- | --- |
|  | 2014 | 2015 | 2016 | 2017 | 2018 | 2019 |
|  | Mean (SD) | Mean (SD) | Mean (SD) | Mean (SD) | Mean (SD) | Mean (SD) |
| Crosswalks | 12.39 (15.53) | 12.67 (21.27) | 14.92 (20.72) | 10.38 (13.94) | 10.68 (11.58) | 9.66 (8.65) |
| Sidewalks | 64.12 (24.16) | 70.39 (28.79) | 67.80 (23.66) | 62.77 (27.89) | 59.87 (18.76) | 60.83 (15.50) |
| Streetlights | 34.85 (21.10) | 35.57 (27.04) | 39.75 (24.76) | 33.33 (22.29) | 32.26 (15.56) | 32.55 (13.90) |
| Stop signs | 2.58 (4.82) | 2.03 (5.85) | 2.51 (5.19) | 2.21 (4.07) | 2.26 (3.30) | 2.09 (1.58) |
| N | 162 | 143 | 152 | 155 | 166 | 169 |

**Figure S1.** Correlations between built environment characteristics and census tract characteristics, 2019. Data sources: Google Street View images (built environment characteristics) and the American Community Survey (census tract sociodemographic characteristics).


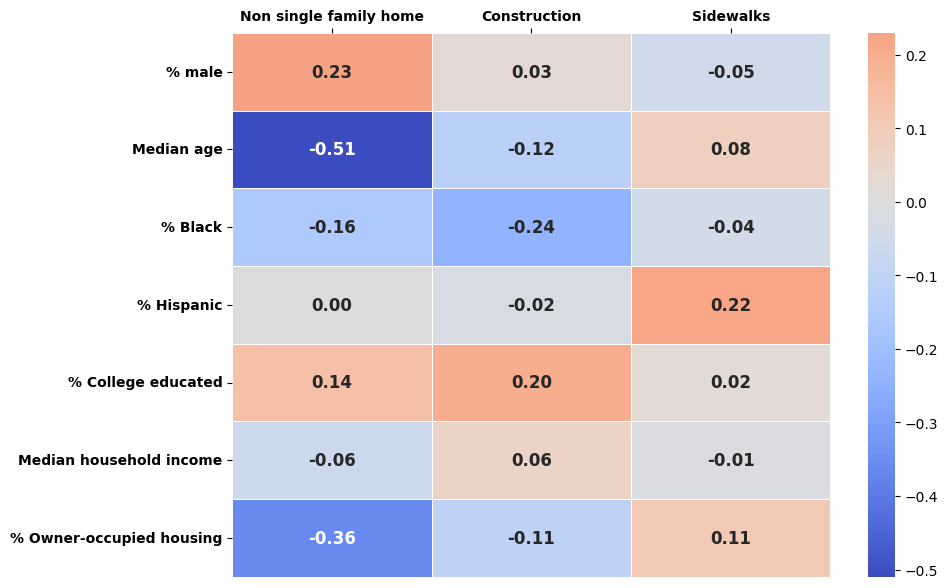


| **Table S2.** Built environment predictors of adult health outcomes.^a^ | | | | | | |
| --- | --- | --- | --- | --- | --- | --- |
|  | Obesity | Diabetes | High blood pressure | High cholesterol | Cancer | Mental Health |
| *Predictors* | % (95% CI)^b^ | % (95% CI)^b^ | % (95% CI)^b^ | % (95% CI)^b^ | % (95% CI)^b^ | % (95% CI)^b^ |
| Year (centered 2016) | 0.11 (0.03, 0.18) | **-0.07 (-0.11, -0.02)** | **0.13 (0.07, 0.19)** | **-1.18 (-1.25, -1.11)** | **0.06 (0.05, 0.08)** | **0.75 (0.68, 0.82)** |
| Single lane road | 0.10 (-0.04, 0.24) | 0.02 (-0.06, 0.09) | 0.01 (-0.04, 0.07) | 0.03 (-0.07, 0.12) | -0.01 (-0.05, 0.02) | -0.01 (-0.12, 0.09) |
| Stop signs | 0.00 (-0.10, 0.10) | 0.00 (-0.05, 0.06) | 0.00 (-0.04, 0.04) | -0.01 (-0.08, 0.06) | 0.01 (-0.02, 0.03) | 0.00 (-0.07, 0.08) |
| 2 or more cars | -0.08 (-0.26, 0.11) | -0.05 (-0.15, 0.06) | **-0.12 (-0.19, -0.04)** | -0.11 (-0.24, 0.02) | 0.01 (-0.03, 0.06) | 0.04 (-0.10, 0.17) |
| Streetlights | 0.09 (-0.06, 0.25) | 0.03 (-0.05, 0.12) | 0.01 (-0.05, 0.07) | 0.00 (-0.11, 0.11) | -0.02 (-0.05, 0.02) | 0.02 (-0.09, 0.14) |
| Not single-family home | **-0.19 (-0.36, -0.02)** | -0.08 (-0.17, 0.02) | -0.03 (-0.10, 0.04) | 0.04 (-0.07, 0.16) | 0.00 (-0.04, 0.04) | 0.07 (-0.06, 0.19) |
| Sidewalks | 0.02 (-0.16, 0.21) | 0.02 (-0.08, 0.12) | 0.05 (-0.02, 0.13) | **0.16 (0.03, 0.29)** | -0.03 (-0.07, 0.02) | -0.11 (-0.25, 0.03) |
| Construction | -0.07 (-0.21, 0.07) | -0.05 (-0.13, 0.03) | 0.02 (-0.04, 0.08) | -0.07 (-0.17, 0.03) | -0.02 (-0.05, 0.02) | -0.04 (-0.15, 0.07) |
| *Census tract sociodemographic* | |  |  |  |  |  |
| Percent male | 0.01 (-0.19, 0.22) | -0.12 (-0.24, 0.01) | -0.13 (-0.25, 0.00) | **-0.22 (-0.40, -0.04)** | -0.01 (-0.06, 0.05) | 0.09 (-0.08, 0.26) |
| Median age | -0.19 (-0.46, 0.08) | **0.36 (0.19, 0.52)** | 0.09 (-0.08, 0.26) | **0.39 (0.14, 0.64)** | **0.08 (0.00, 0.15)** | **-0.56 (-0.78, -0.34)** |
| Percent Black | **6.66 (6.19, 7.12)** | **3.70 (3.40, 3.99)** | 3.96 (3.38, 4.54) | **3.01 (2.45, 3.56)** | 0.05 (-0.13, 0.23) | **2.52 (2.15, 2.88)** |
| Percent Hispanic | **0.67 (0.40, 0.93)** | **0.39 (0.23, 0.56)** | **0.62 (0.44, 0.80)** | **0.67 (0.41, 0.93)** | -0.03 (-0.11, 0.05) | 0.14 (-0.07, 0.35) |
| Percent college educated | **0.45 (0.26, 0.64)** | **0.22 (0.11, 0.33)** | **0.34 (0.26, 0.43)** | **0.55 (0.41, 0.69)** | **-0.06 (-0.10, -0.01)** | 0.12 (-0.03, 0.27) |
| Median household income | **-0.86 (-1.28, -0.43)** | **-0.55 (-0.80, -0.29)** | **-0.68 (-0.93, -0.43)** | 0.23 (-0.13, 0.60) | -0.01 (-0.12, 0.10) | -0.11 (-0.46, 0.23) |
| Percent owner-occupied housing | **-0.77 (-1.15, -0.39)** | -0.09 (-0.33, 0.15) | -0.29 (-0.62, 0.04) | 0.10 (-0.31, 0.52) | **0.29 (0.17, 0.42)** | **-1.14 (-1.44, -0.84)** |
| ^a^Data source for health outcome: CDC PLACES. | | | | | | |
| ^b^Multilevel mixed effects generalized linear models were run for each outcome separately with random intercepts at the year (2013-2019) and census tract levels (921 tracts). Models controlled for census tract median age, percent male, percent Hispanic, percent black, percent with a college degree, median household income, and percent owner-occupied housing. Built environment characteristics were analyzed as continuous variables. All variables were standardized to have a mean value of 0 and a standard deviation of 1, to enable comparisons across health outcomes. | | | | | | |
| * p<0.05 † p<0.10. |  |  |  |  |  |  |
